# A Review of the Inhibition of the Mitochondrial ATP Synthase by IF1 *in vivo*: Reprogramming Energy Metabolism and Inducing Mitohormesis

**DOI:** 10.3389/fphys.2018.01322

**Published:** 2018-09-19

**Authors:** Ana García-Aguilar, José M. Cuezva

**Affiliations:** Departamento de Biología Molecular, Centro de Biología Molecular Severo Ochoa (CSIC-UAM), Centro de Investigación Biomédica en Red de Enfermedades Raras CIBERER-ISCIII, Instituto de Investigación Hospital 12 de Octubre (i+12), Universidad Autónoma de Madrid, Madrid, Spain

**Keywords:** ATP synthase, ATPase inhibitory factor 1, energy metabolism, metabolic preconditioning, mitohormesis, reactive oxygen species, stress kinases, transgenic mice

## Abstract

The ATPase Inhibitory Factor 1 (IF1) is the physiological inhibitor of the mitochondrial ATP synthase. Herein, we summarize the regulation of the expression and activity of IF1 as a main driver of the activity of oxidative phosphorylation (OXPHOS) in mammalian tissues. We emphasize that the expression of IF1, which is a mitochondrial protein with very short half-life, is tissue-specifically expressed and primarily controlled at posttranscriptional levels. Inhibition of the activity of IF1 as inhibitor of the ATP synthase under normal physiological conditions is exerted by phosphorylation of S39 by a cAMP-dependent PKA-like activity of mitochondria in response to different physiological cues. Conditional tissue-specific transgenic mice overexpressing IF1 in colon, or a mutant active version of IF1 (IF1-H49K) in liver or in neurons, revealed the inhibition of the ATP synthase and the reprograming of energy metabolism to an enhanced glycolysis. In the IF1-H49K models, the assembly/activity of complex IV and the superassembly of complex V are also affected. Moreover, the IF1-mediated inhibition of the ATP synthase generates a reactive oxygen species (mtROS) signal that switches on the expression of nuclear genes that facilitate adaptation to a restrained OXPHOS. In contrast to normal mice, metabolically preconditioned animals are partially protected from the action of cytotoxic agents by upgrading the activation of stress kinases and transcription factors involved in resolving metabolic adaptation, the antioxidant response, cell survival, and the immune response of the tissue microenvironment. Altogether, we stress a fundamental physiological function for the ATP synthase and its inhibitor in mitohormesis.

## Introduction

The ATP synthase is the multi-subunit membrane-bound enzyme of mitochondria that utilizes the proton electrochemical gradient generated by the respiratory chain for the synthesis of ATP (Walker, 2013). The activity of the ATP synthase regulates the flux of oxidative phosphorylation (OXPHOS; Boyer, 1997), the execution of cell death (Bernardi et al., 2015), and mitochondrial signaling by reactive oxygen species (ROS; Formentini et al., 2012; Martinez-Reyes and Cuezva, 2014). The ATP synthase has an inner membrane embedded domain known as F_O_ (subunits *c*_8-10_ *a*_1_) and a membrane extrinsic domain called F1 (subunits α_3_β_3_γδε). The two domains are linked together by a central stalk, composed of subunits γ, δ, and ε of the F1 sector, and a peripheral stalk composed of subunits *b*, *d*, *f*, A6L, F_6_, and OSCP (Walker, 2013). The enzyme also incorporates other minor subunits in the membrane embedded sector (Walker, 2013; He et al., 2018). The F1 domain is the catalytic core of the enzyme and it connects the synthesis or hydrolysis of ATP to proton transport through the Fo domain (Boyer, 1997; Walker, 2013). The structures of the two domains of the ATP synthase have been solved by X-ray crystallography (Abrahams et al., 1994; Stock et al., 1999). Recently, the high-resolution structures of yeast mitochondria and chloroplast ATP synthases have been determined by cryo-electron microscopy (Hahn et al., 2018; Srivastava et al., 2018).

The ATPase Inhibitory Factor 1 (IF1) is a physiological inhibitor of the enzyme (Pullman and Monroy, 1963). The mammalian IF1 is a small structurally disordered nuclear-encoded mitochondrial protein (Gledhill et al., 2007; Esparza-Molto et al., 2017). Upon import of the precursor protein into mitochondria, its pre-sequence is cleaved to render the mature IF1, a α-helical protein that contains the inhibitory domain at the N-terminus and a dimerization domain at the C-terminus. The structure of an inhibitory peptide (I1-60His) lacking most of the dimerization domain of bovine IF1 has been solved bound to the F1 domain of the ATP synthase (Gledhill et al., 2007; Bason et al., 2014). The docking site of the I1-60His peptide is a groove between the α-helices of α and β subunits of the F1 domain reaching the γ subunit of the central stalk with its N-terminus (Gledhill et al., 2007). *In vitro*, IF1 interacts with the ATP synthase in a 1:1 ratio at a pH of 6.7 and below (Cabezon et al., 2000, 2001). From these and other findings (Campanella et al., 2008), it has been suggested that IF1 exerts its biological function to prevent the hydrolysis of ATP by reverse functioning of the enzyme under mitochondrial depolarizing conditions such as in hypoxia (Campanella et al., 2008; Walker, 2013; Garcia-Bermudez and Cuezva, 2016). However, recent findings indicate that IF1 inhibits both the synthase and hydrolase activity of the ATP synthase (Sanchez-Cenizo et al., 2010; Garcia-Bermudez et al., 2015). In this mini review, we will briefly summarize recent findings regarding the regulation of the expression and activity of IF1, playing special emphasis to the results obtained in conditional tissue-specific transgenic mice overexpressing IF1 or an active mutant version of the protein. More detailed reviews on other aspects in this subject can be found elsewhere (Garcia-Bermudez and Cuezva, 2016; Esparza-Molto et al., 2017; Esparza-Moltó and Cuezva, 2018).

## Posttranscriptional Regulation of IF1 Expression

**FIGURE 1:**
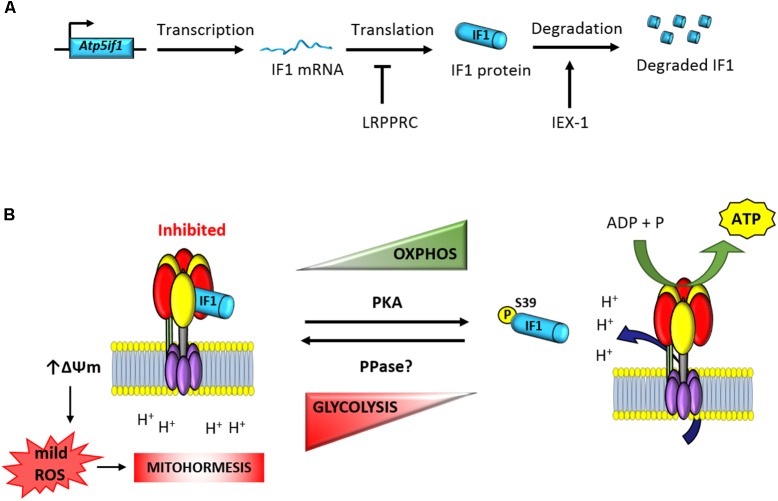
Posttranscriptional regulation of the expression and activity of IF1. **(A)** The expression of the short-lived IF1 protein is tissue-specific and regulated at posttranscriptional levels (Sanchez-Arago et al., 2013b,c). The mRNA binding protein LRPPRC participates in the negative control of IF1 expression (Mourier et al., 2014). Moreover, IEX-1 interacts with IF1 to target the protein to degradation (Shen et al., 2009). **(B)** The phosphorylation of IF1 by PKA-mediated prevents its binding to the ATP synthase and allows efficient synthesis of ATP (Garcia-Bermudez et al., 2015). In contrast, dephosphorylated IF1 binds the ATP synthase and inhibits ATP synthesis (Garcia-Bermudez et al., 2015). Inhibition of the enzyme triggers the raise in mitochondrial membrane potential and the production of mitochondrial ROS (Sanchez-Cenizo et al., 2010). mtROS act as second messengers to signal in the nucleus mitohormetic responses. *Atp5if1*, mouse gene of ATP synthase inhibitory factor 1; LRPPRC, leucine-rich pentatricopeptide repeat motif-containing protein; IEX-1, immediate early response X-1; PKA, protein kinase A; PPase, protein phosphatase; ΔΨm, mitochondrial membrane potential.

Based on *in situ* hybridization databases, it has been suggested that IF1 is ubiquitously expressed in mammalian tissues (Faccenda et al., 2017). However, analysis of the expression of the protein in several human tissues revealed that its expression varies greatly, with high expression restricted to heart, liver, kidney, stomach, and brain and negligible expression in breast, colon, lung, and ovary (Sanchez-Cenizo et al., 2010; Sanchez-Arago et al., 2013b). In contrasts, IF1 is highly overexpressed in human colon, lung, breast and ovarian carcinomas (Sanchez-Cenizo et al., 2010; Sanchez-Arago et al., 2013b; Esparza-Molto et al., 2017) and in cancer cell lines (Sanchez-Cenizo et al., 2010). Interestingly, the overexpression of IF1 in these carcinomas is exerted in the absence of relevant changes in the expression levels of IF1 mRNA (Sanchez-Arago et al., 2013b). Likewise, the large differences noted in the expression of IF1 between hMSCs and differentiated osteocytes also happen in the absence of cellular changes in the availability of IF1 mRNA (Sanchez-Arago et al., 2013c). Consistent with a relevant role played by IF1 in cellular metabolic reprograming, pulse-chase (Sanchez-Arago et al., 2013b) and protease inhibitor (Shen et al., 2009; Sanchez-Arago et al., 2013b,c) experiments have shown that IF1 is a short-lived mitochondrial protein with a half-life of ∼2 h (Sanchez-Arago et al., 2013b). Moreover, changes in the expression of IF1 during cellular differentiation are accompanied by profound changes in the rate of IF1 turnover (Sanchez-Arago et al., 2013c). Hence, these findings support that regulation of IF1 expression is tissue-specific and controlled at posttranscriptional levels (**Figure 1A**). Studies addressing the basal expression and mechanisms that regulate IF1 levels in human and mouse tissues are required in order to deepen into the characterization of its role in cellular physiology and in the regulation of the ATP synthase.

The protein encoded in the stress-inducible mouse IEX-1 (Immediate Early response gene X-1) gene has been shown to regulate the cellular expression level of IF1 in CHO cells (Shen et al., 2009). Overexpression of IEX-1 resulted in the downregulation of IF1 by targeting the protein to degradation by an unidentified protease (**Figure 1A**; Shen et al., 2009). However, the tumor suppressor IER3 (Sebens Muerkoster et al., 2008), which is the human homolog of IEX-1, is overexpressed in human carcinomas that display high expression levels of IF1 (Sanchez-Arago et al., 2013b). Moreover, partial silencing of IER3 in colon cancer cells has no relevant effect on IF1 expression levels (Sanchez-Arago et al., 2013b), raising the intriguing possibility that the control of the stability of the protein might be species-specific and cell-type regulated. A short-interfering RNA-based screen aimed at the identification of potential proteases involved in the degradation of IF1 in human colon cancer cells and hMSCs failed to provide candidates for the degradation of IF1 (Sanchez-Arago et al., 2013c), suggesting a complex regulation of the mechanisms involved in its degradation.

Consistent with the stringent tissue-specific posttranscriptional regulation of IF1 expression, it has been described that conditional knockout mice devoid of the RNA binding protein LRPPRC (leucine rich pentatricopeptide repeat containing protein) in heart have impaired ATP synthase activity as a result of a deficient assembly of the enzyme (Mourier et al., 2014). Interestingly, the upregulation of IF1 in heart of these mice takes place in the absence of relevant changes in the availability of IF1 mRNA and it is accompanied by the appearance of IF1-bound in subassembled ATP synthase complexes (Mourier et al., 2014). Similarly, knockdown of LRPPRC in mouse liver also compromises ATP synthase activity by deficient assembly of the enzyme (Cuillerier et al., 2017). Knockdown of LRPPRC produces a generalized assembly defect in OXPHOS complexes containing mtDNA-encoded subunits, due to a severe decrease in mitochondrial mRNAs (Gohil et al., 2010; Sasarman et al., 2010). Moreover, it is known that LRPPRC associates with both nuclear and mitochondrial mRNAs and as such is a candidate for coordinating nuclear and mitochondrial gene expression at posttranscriptional levels (Mili and Pinol-Roma, 2003). Hence, it is possible that LRPPRC could bind and participate in the control of the translation of the nuclear encoded IF1 mRNA (**Figure 1A**). In this situation, the absence of LRPPRC might contribute to the upregulation of the IF1 protein observed in the heart of the LRPPRC knockout mice either by increasing the stability of the protein and/or the rate of IF1 mRNA translation, a suggestion that deserves further investigation.

Remarkably, recent findings have stressed the role of IF1 bound to the key inhibited intermediate F1-c8 complex in the assembly pathway of the mammalian ATP synthase (He et al., 2018). Hence, it seems reasonable to suggest that the increased abundance of IF1 in heart of LRPPRC (−/−) mice could also contribute to impair mitochondrial ATP synthase assembly by interfering, perhaps by mass-action ratio, its release from the assembly intermediate in the late stage in ATP synthase assembly (He et al., 2018). This suggestion is in agreement with the appearance of IF1-bound in subassemblies of ATP synthase complexes (Mourier et al., 2014; Cuillerier et al., 2017). It seems obvious that additional studies are required to unveil the posttranscriptional mechanisms that regulate the tissue-specific biogenesis of mitochondrial OXPHOS complexes and specifically of those affecting the ATP synthase and its inhibitor, both in health and in disease.

## Regulation of IF1 Activity by Phosphorylation

Most studies have emphasized the role of IF1 as a unidirectional inhibitor of the reverse functioning of the ATP synthase (Walker, 2013); that is, as inhibitor of ATP hydrolysis upon mitochondrial depolarization and/or at an acidic mitochondrial pH (for recent reviews, see Garcia-Bermudez and Cuezva, 2016; Esparza-Molto et al., 2017). The pH regulates the ionization state of histidines placed in the C-terminal α-helix of the protein influencing IF1 oligomerization (Cabezon et al., 2000, 2001; Campanella et al., 2008). Above neutral pH, IF1 forms inactive tetramers by occlusion of its inhibitory N-terminal disordered domain. One important residue of IF1 is histidine 49, being the mutant H49K protein active as inhibitor even at pH above neutrality (Schnizer et al., 1996). This mutant has been used to inhibit OXPHOS *in vivo* in transgenic mice (Formentini et al., 2014; Santacatterina et al., 2016) and will be commented in the next section of this review.

In addition to the pH regulated activity of IF1, we have recently described that the regulation of its activity as inhibitor of the ATP synthase also involves protein phosphorylation (**Figure 1B**; Garcia-Bermudez et al., 2015; Garcia-Bermudez and Cuezva, 2016). In fact, the development and expression of the phospho-deficient and phospho-mimetic serine mutants of IF1 demonstrated that phosphorylation of IF1 in serine 39 (S39) renders a protein unable to bind the ATP synthase what results in an increase of the enzyme activity (**Figure 1B**; Garcia-Bermudez et al., 2015). Only the dephosphorylated IF1 or the phospho-deficient S39A is able to bind and inhibit both the synthase and hydrolase activities of the enzyme in different physiological situations that result in the reprogramming of cellular energy metabolism to an enhanced glycolysis (**Figure 1B**; Garcia-Bermudez et al., 2015; Garcia-Bermudez and Cuezva, 2016). In this regard, dephosphorylated IF1 is present in hypoxic cells and the fraction of IF1 bound to the ATP synthase is significantly increased in this situation (Garcia-Bermudez et al., 2015). Likewise, IF1 is also found dephosphorylated in cells progressing through the reductive phase of the cell cycle (S/G2/M; Garcia-Bermudez et al., 2015). Interestingly, metabolic reprogramming to an enhanced aerobic glycolysis in human carcinomas also correlates with the expression of dephosphorylated IF1 (Garcia-Bermudez et al., 2015). In contrast, cells in G1, the high-energy demanding phase of the cell cycle, display phosphorylated IF1 and an increased ATP synthase activity (Garcia-Bermudez et al., 2015).

The phosphorylation status of IF1 depends on the cell type analyzed (Garcia-Bermudez et al., 2015). An increased phosphorylation of IF1 is found in cells stimulated with the adenylate cyclase activator forskolin and with db-cAMP, the membrane permeable activator of PKA (**Figure 1B**; Garcia-Bermudez et al., 2015). In contrast, the inhibition of PKA activity with different of its inhibitors (H89 and PKI) is related with the dephosphorylation of IF1 and the inhibition of the ATP synthetic activity of the enzyme (Garcia-Bermudez et al., 2015). The relevance of the cAMP/PKA signaling pathway in controlling the phosphorylation of IF1 and subsequent activation of the ATP synthase activity was further demonstrated *in vivo* by treatment of mice with β-adrenergic effectors (Garcia-Bermudez et al., 2015). Administration of the agonist clenbuterol promoted a significant increase in the fraction of phosphorylated IF1 present in heart concurrently with an increase in the ATP synthase activity in mitochondria. In contrast, administration of the β-adrenergic antagonist propranolol had the opposite effects (Garcia-Bermudez et al., 2015). These findings also suggested that in some high-energy demanding tissues, there is a fraction of IF1-bound to the enzyme, maintaining a pool of inactive ATP synthase in order to facilitate the tissue response to a sudden physiological increase in energy requests (Garcia-Bermudez et al., 2015). Clearly, there is lack of knowledge regarding the tissue content and state of phosphorylation of IF1 in relation to the content of the ATP synthase in mammalian tissues. We think this is a critical issue to explain the tissue-specific regulation of OXPHOS in pathophysiology.

PKA is activated by cAMP and is known to regulate the efficiency of OXPHOS (Acin-Perez et al., 2009, 2011; Di Benedetto et al., 2013; Garcia-Bermudez and Cuezva, 2016). The synthesis of cAMP is compartmentalized (Di Benedetto et al., 2013) and could be exerted by the activation of plasma membrane or soluble adenylyl cyclases (sACs; Ould Amer and Hebert-Chatelain, 2018). A sAC has been described inside mitochondria (Acin-Perez et al., 2009; Di Benedetto et al., 2014; Lefkimmiatis and Zaccolo, 2014), which is activated by Ca^2+^ (Di Benedetto et al., 2013) and bicarbonate (Acin-Perez et al., 2009). In heart, upon the activation of G-protein coupled receptors by β-adrenergic agonists, cytoplasmic Ca^2+^ increases to activate muscle contraction which is supported by the ATP produced by the Ca^2+^-mediated activation of OXPHOS (Glancy and Balaban, 2012). Hence, we have suggested that the activation of the mitochondrial sAC in response to the mitochondrial sequestration of Ca^2+^ (Rizzuto et al., 2012) could be a key element in promoting the intramitochondrial rise in cAMP concentrations observed in response to clenbuterol administration (Garcia-Bermudez et al., 2015). In this situation, IF1 could be phosphorylated by the activity of an intramitochondrial cAMP-dependent protein kinase A like activity rendering an enhanced production of ATP by OXPHOS (Garcia-Bermudez and Cuezva, 2016). However, despite PKA has been described in mitochondria (Pagliarini and Dixon, 2006; Sardanelli et al., 2006; Garcia-Bermudez and Cuezva, 2016), the mitochondrial site of PKA action is still a matter of debate (Monterisi et al., 2017; Ould Amer and Hebert-Chatelain, 2018). In this situation, we cannot rule out that PKA-anchored to one of the three major A-kinase anchor proteins (AKAPs) that target PKA to the outer mitochondrial membrane (Garcia-Bermudez et al., 2015; Garcia-Bermudez and Cuezva, 2016) could phosphorylate IF1 prior to its import into the organelle. In fact, that would be in agreement with the observed phosphorylation of IF1 in response to treatment of the cells with forskolin (Garcia-Bermudez et al., 2015), which is an activator of the transmembrane adenylyl cyclase. Examples of mitochondrial proteins phosphorylated by the activity of PKA in advance of its import are already available (De Rasmo et al., 2008).

Attenuation of the cAMP signal on targeted proteins is exerted by the large family of phosphodiesterases (Maurice et al., 2014) that degrade the localized pool of cAMP (Acin-Perez et al., 2011; Monterisi et al., 2017) and by the activity of protein phosphatases (Lim et al., 2016; Ould Amer and Hebert-Chatelain, 2018). The mechanisms regulating the dephosphorylation of IF1 are presently unknown.

Overall, IF1 inhibits both the synthetic and hydrolytic activities of the ATP synthase as long as the inhibitor protein is bound to the enzyme (Garcia-Bermudez et al., 2015). The binding of IF1 to the ATP synthase is a physiologically regulated process in which the phosphorylation status of IF1 plays a prominent role (Garcia-Bermudez et al., 2015). Under pathophysiological conditions triggered by a mitochondrial deficiency in oxygen availability and matrix acidification, it has been suggested that IF1 can bind the ATP synthase to prevent its hydrolase activity (Rouslin and Pullman, 1987; Campanella et al., 2008; Walker, 2013). However, recent findings argue against the operation of the ATP synthase in reverse under hypoxic conditions (Sgarbi et al., 2018). In fact, these authors have shown that the hydrolase activity of the ATP synthase is not operative unless mitochondria are challenged by the addition of an uncoupler, an extreme anoxia-mimicking condition (Sgarbi et al., 2018). In addition, it should be taken into consideration that any biochemical manifestation of the effect of IF1 on ATP synthase activities largely depend on the molar ratio that exists between the inhibitor protein and the ATP synthase in the mitochondria of that particular cell, because the mass-action ratio also controls the interaction of both proteins (Sanchez-Cenizo et al., 2010).

## Overexpression of If1 in Tissue-Specific Conditional Transgenic Mice

**FIGURE 2:**
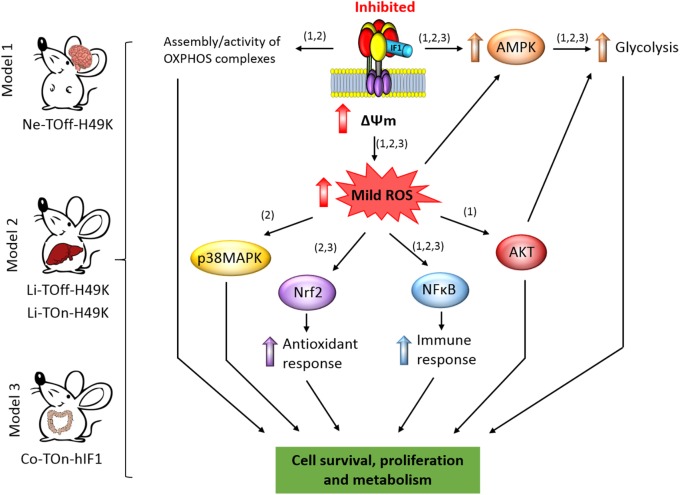
IF1-mediated inhibition of the ATP synthase and signaling pathways of mitohormesis. The scheme shows the conditional tissue-specific transgenic mouse models developed and summarized in this mini review. The transgenic mice expressing the human IF1-H49K in brain (Formentini et al., 2014) or in liver (Santacatterina et al., 2016) or IF1 in the colon (Formentini et al., 2017) reprogram energy metabolism to an enhanced glycolysis by the inhibition of the ATP synthase. In the mouse models expressing IF1-H49K, an effect of the transgene is manifested in the assembly/activity of some OXPHOS complexes (Formentini et al., 2014; Santacatterina et al., 2016). The mitohormetic response involves the activation of the indicated stress kinases and transcription factors in response to the generation of mtROS by mitochondrial hyperpolarization (ΔΨm) as a result of the inhibition of the ATP synthase. Overall, the main effect of mitohormesis is to warranty cell survival, metabolism, the antioxidant, and immune responses of the organism to allow adaptation to different stressful conditions. The numbers in brackets indicate the corresponding mouse model. AMPK, AMP-activated protein kinase; p38MAPK, mitogen activated protein kinase p38; AKT, protein kinase B; NFB, nuclear factor kappa B; Nrf2, nuclear factor (erythroid-derived 2)-like 2.

Several findings fostered the idea that IF1, besides regulating the production of ATP, exerts additional functions in mitochondrial physiology (Bernardi et al., 2015; Esparza-Molto et al., 2017). In fact, inhibition of the activity of the ATP synthase by IF1 is known to trigger an increase in mitochondrial membrane potential by preventing the backflow of H ^+^ into mitochondria generating the subsequent production of reactive oxygen species in mitochondria (mtROS; **Figure 1B**; (Sanchez-Cenizo et al., 2010; Formentini et al., 2012; Sanchez-Arago et al., 2013b). It is accepted that the amount and site of production of mtROS, which are essential signaling molecules, defines the nuclear response of the cell to different cues (Martinez-Reyes and Cuezva, 2014; Yun and Finkel, 2014; Shadel and Horvath, 2015). This process, coined as retrograde signaling from mitochondria to the nucleus, finally determines cellular responses by controlling the expression of nuclear genes that facilitate adaptation of the organism to different physiological cues or cytotoxic agents (Quiros et al., 2016; Esparza-Molto et al., 2017). In this regard, there is growing evidence supporting that a mild mitochondrial stress can protect cells from subsequent insults, a concept termed mitohormesis (**Figure 1B**; Ristow, 2014; Yun and Finkel, 2014; Chandel, 2015; Quiros et al., 2016; Esparza-Molto et al., 2017), because of the activation of cytoprotective mechanisms that are induced to compensate the first insult. The induction of mitohormesis can be accomplished by different ways that affect mitochondrial function such as by inhibitors of the electron transport chain, mitochondrial translation, mtROS generators, etc. Eliciting mitohormetic responses can impact on both an increase in organismal lifespan and/or an improved health. In the following, we will review the mitohormetic responses affecting health in transgenic mice by overexpressing IF1 to inhibit *in vivo* the activity of OXPHOS in neurons (Formentini et al., 2014), hepatocytes (Santacatterina et al., 2016), and colonocytes (Formentini et al., 2017; **Figure 2**). Mitohormetic responses targeting the ATP synthase and affecting lifespan have been recently reviewed elsewhere (Esparza-Molto et al., 2017).

### Overexpression of IF1-H49K in Neurons

Mice expressing the pH-insensitive constitutively active mutant H49K version of human IF1 (IF1-H49K) in neurons (**Figure 2**) revealed the inhibition of the ATP synthase as assessed in total brain extracts, isolated brain mitochondria, and primary cultures of cortical neurons without affecting the expression of proteins from different OXPHOS complexes (Formentini et al., 2014). Consistent with the inhibition of the synthase, transgenic mice revealed a significant reduction in brain ATP concentrations, the activation of the metabolic sensor AMPK (**Figure 2**) and the concurrent increased expression of glycolytic proteins (Formentini et al., 2014). Interestingly, and when compared to control littermates, the inhibition of the ATP synthase also resulted in the inhibition of the activity of Complex IV of the respiratory chain by preventing the assembly of monomers of Complex IV into supercomplexes (Formentini et al., 2014). Primary cultures of cortical neurons of transgenic mice confirmed the IF1-mediated inhibition of the ATP synthase, the functional and proteomic reprogramming of neurons to an enhanced aerobic glycolysis and importantly, the increased production of mtROS and carbonylation of cellular proteins when compared to neurons of control littermates (Formentini et al., 2014). Despite the changes observed in transgenic mice, we noted no major phenotypic differences when compared to controls unless the animals were challenged by a cytotoxic insult (Formentini et al., 2014).

Phenotypic analysis of mice after injection of quinolinic acid into the left striatal region of the brain to induce neurotoxicity (Schwarcz et al., 2012) revealed that transgenic mice had less neuronal death and gliosis when compared to control littermates (Formentini et al., 2014). In other words, transgenic mice were partially protected from damage, indicating that metabolic preconditioning afforded by the inhibition of the ATP synthase protects neurons from the oxidative insult (**Figure 2**). This finding that was also confirmed in cultures of cortical neurons primed to death by glutamate addition (Formentini et al., 2014). Three different locomotor tests further confirmed the better performance of transgenic mice over controls in neurological examinations, also coinciding with a better maintenance of the cellular redox state in the affected hemisphere of mice expressing IF1-H49K (Formentini et al., 2014). Analysis of the signaling pathways involved in cell survival revealed that brain extracts of metabolically preconditioned mice showed and increased activation of the Akt/mTORC1 and NFκB pathways concurrently with the activation of PARP repair mechanisms (**Figure 2**; Formentini et al., 2014). Neuronal protection appeared to result from mtROS activation of pro-survival pathways against oxidative stress because quenching mtROS with the ROS scavenger MitoQ ameliorated glutamate-induced cell death of cortical neurons (Formentini et al., 2014). Overall, the metabolic stress imposed in neurons by partial IF1-mediated inhibition of the ATP synthase unleashed a mitohormetic response that helped to overcome the deleterious effects of a neurotoxic agent (**Figure 2**; Formentini et al., 2014).

### Overexpression of IF1-H49K in Hepatocytes

We have also developed transgenic Tet-On and Tet-Off mice that express the IF1-H49K transgene in the hepatocytes (**Figure 2**; Santacatterina et al., 2016). In both models, the expression of IF1-H49K promoted the inhibition of the ATP synthase as assessed by the partial inhibition of respiration in isolated liver mitochondria and the reduction of liver ATP concentrations, without affecting the expression of proteins from different OXPHOS complexes (Santacatterina et al., 2016). Consistent with the inhibition of the ATP synthase, transgenic mice revealed the activation of the stress kinases AMPK and p38 MAPK (**Figure 2**) and developed hypoglycemia upon overnight starvation when compared to controls (Santacatterina et al., 2016). Interestingly, as in the case of the brain model (Formentini et al., 2014), the IF1-mediated inhibition of liver ATP synthase *in vivo* also resulted in the inhibition of the activity of Complex IV of the respiratory chain by impeding its assembly into supercomplexes (Santacatterina et al., 2016). Moreover, transgenic mice expressing IF1-H49K also showed a higher abundance of dimers of the ATP synthase (Santacatterina et al., 2016), exhibited the induction of superoxide dismutase (SOD2) and a less basal carbonylation of liver proteins than controls (Santacatterina et al., 2016). Despite these phenotypic differences, 1-year follow-up of the animals revealed no differences in weight, lifespan, and cage behavior unless they were challenged by a cytotoxic agent (Santacatterina et al., 2016).

In the case of the liver model we used acetaminophen (APAP) to induce cell death (Santacatterina et al., 2016). APAP is hepatotoxic causing acute liver failure by inducing mitochondrial dysfunction and oxidative stress (Nelson et al., 2015). The results obtained after administration of APAP stressed that livers of mice overexpressing IF1-H49K had much lower rates of cell death and a lesser oxidative damage of liver of proteins than littermate controls (Santacatterina et al., 2016), supporting that they are partially protected from the oxidative insult (**Figure 2**; Santacatterina et al., 2016). Interestingly, and upon APAP administration, the nuclear factor-erythroid 2-related factor (Nrf2)-guided antioxidant response was strongly induced in hepatocytes from mice overexpressing IF1-H49K (**Figure 2**; Santacatterina et al., 2016). Likewise, the NFκB survival pathway was also preferentially induced in the livers of transgenic mice (**Figure 2**; Santacatterina et al., 2016). Overall, the message from the mouse model overexpressing the IF1-H49K mutant is that the metabolic reprogramming imposed in hepatocytes by partial inhibition of the ATP synthase developed a mitohormetic response that helped to overcome the deleterious effects of the APAP (Santacatterina et al., 2016).

### Overexpression of IF1 in the Intestine

The third tissue-specific transgenic mouse model that we have generated and studied is that expressing the wild-type version of human IF1 in the intestine (Formentini et al., 2017). Mice overexpressing IF1 revealed the inhibition of the ATP synthase activity in colonocytes (Formentini et al., 2017). In contrast to the previously described mouse models, under basal conditions, we observed no significant reduction in tissue ATP concentrations and in the activation of the stress kinases AMPK, Akt, and p38 MAPK in the colon of IF1 expressing mice when compared to controls (**Figure 2**; Formentini et al., 2017). However, metabolic reprograming of the tissue to an enhanced glycolysis was evidenced by an increased expression of several glycolytic proteins in colon extracts. Moreover, isolated colonocytes of transgenic mice showed the reduction of ADP-stimulated respiration concurrently with an increased production of lactate when compared to controls, consistent with the inhibition of the ATP synthase by the overexpression of IF1 (Formentini et al., 2017). Remarkably, and under basal conditions, the colon of transgenic mice showed a sharp induction of the canonical NFκB pathway (**Figure 2**) that paralleled an enhanced carbonylation of tissue proteins, suggesting that mtROS generated in response to the IF1-mediated inhibition of the ATP synthase activated the master regulator of the inflammatory response (Karin, 2006). Interestingly, activation of the NFκB pathway in the intestine of transgenic mice triggered the induction of an anti-inflammatory phenotype in plasma and in the colon as evidenced by the analysis of markers of immune cell populations, cytokines and other immune-regulatory NFκB targeted genes (Formentini et al., 2017). However, as previously noted with the two other transgenic mouse models, follow-up of the animals revealed no relevant differences unless the animals were challenged by a cytotoxic agent (Formentini et al., 2017).

To explore the potential anti-inflammatory phenotype induced by the overexpression of IF1 (Formentini et al., 2017), we followed a standard protocol of DSS-induced colitis (Wirtz et al., 2007). Despite DSS-induced colitis produced severe oxidative damage, cell death, and inflammation in both control and IF1-expressing mice, the effects were much less pronounced in the transgenic animals (Formentini et al., 2017), supporting that the IF1-mediated metabolic preconditioning provides protection against inflammation. Interestingly, the inflammatory response in control mice was geared by the activation of the pro-inflammatory STAT3 transcription factor and the stress kinases TRIB3, Akt, and the mTOR/p70S6K pathways, whereas transgenic mice showed less or no activation of the former pathways but showed the activation of AMPK (**Figure 2**; Formentini et al., 2017). In this regard, recent findings have stressed the relevance of downregulating the PI3K/AKT/mTOR signaling pathway to ameliorate inflammation (Bai et al., 2018). Protection from inflammation was exerted by the preferential recruitment and polarization of M2 macrophages (F4/80 ^+^ and CD206 ^+^) and Treg cells (CD4 + /FOXP3) – the immune cells that restrain inflammatory responses – in the tissue of transgenic mice when compared to controls (Formentini et al., 2017). Remarkably, quenching mtROS signaling in response to the overexpression of IF1 by the administration of MitoQ to the animals obliterated the induction of the NFκB pathway and the protection from inflammation (Formentini et al., 2017). Similarly, inhibition of NFκB also abolished protection from the inflammatory stress (Formentini et al., 2017). Altogether, supporting that the IF1-mediated mtROS production and the activation of the canonical NFκB pathway play critical roles in immune preconditioning of the colon to favor an anti-inflammatory phenotype of the tissue microenvironment (Formentini et al., 2017). In fact, a deficiency in ROS is known to promote a pro-inflammatory phenotype that favors adenocarcinoma growth after induced colitis (Carvalho et al., 2018). Overall, the message from the mouse model overexpressing IF1 in the intestine is that partial inhibition of the ATP synthase also afforded a mitohormetic response that implicated non-cell autonomous processes by modulating the immune response of the tissue microenvironment.

## Concluding Remarks

We have summarized a large body of the findings that stress that the ATPase IF1 acts as an inhibitor of the ATP synthase activity of the enzyme under normal physiological conditions, contrasting other widely accepted opinions. Inhibition of the ATP synthase compromises the output of ATP by OXPHOS and rewires energy metabolism to an enhanced glycolysis. To understand the regulation of the activity of OXPHOS and eventually its dysregulation in pathology, there are two issues that need to be addressed: first, characterize the tissue-specific expression of the protein, and second, determine the molar ratio that exists between the ATP synthase and its inhibitor in the different human and mouse tissues.

Phosphorylation of S39 in IF1 prevents its binding to the ATP synthase releasing the inhibition of the enzyme. An issue here is to identify the kinases, and perhaps phosphatases, that regulate the activity of IF1 as an inhibitor of the ATP synthase.

The expression of IF1, which is a mitochondrial protein with a very short half-life, is regulated at posttranscriptional levels. RNA binding proteins play a relevant role in controlling the tissue-specific expression of the protein. Importantly, dysregulation of IF1 expression seems to affect the assembly and activity of the ATP synthase and of other OXPHOS complexes. Thus, we need to identify the mechanisms and proteases that participate in controlling the tissue specific expression of IF1. Moreover, the participation of IF1 in the assembly of OXPHOS complexes also deserves additional efforts.

Inhibition of the ATP synthase by IF1 generates a mtROS signal that controls tissue-specific nuclear programs that facilitate the organism to overcome the action of different cytotoxic agents; in other words, induces mitohormesis. As a common denominator of the signaling pathways induced by mitohormesis in the three mouse models, we should highlight AMPK and NFκB (**Figure 2**). Essentially, the IF1-mediated mitohormetic response is expressed by the sharp reduction of cell death in response to the cytotoxic agent. Many studies have highlighted the essential role of the ATP synthase as a critical hub in the execution of cell death (Matsuyama et al., 1998; Santamaria et al., 2006; Sanchez-Arago et al., 2013a). In fact, the ATP synthase is an essential component of the mitochondrial permeability transition pore (PTP; Bonora et al., 2013; Giorgio et al., 2013; Alavian et al., 2014; Bernardi et al., 2015), which is a regulated gate for the efficient execution of cell death (Bernardi et al., 2015; Giorgio et al., 2017; Antoniel et al., 2018). Mitochondrial cristae structure determines the assembly and activity of OXPHOS complexes (Cogliati et al., 2013) and L-optic atrophy 1 (OPA1)-dependent mitochondrial cristae remodeling is a fundamental process in mitochondrial dysfunction gearing cytochrome c release and the production of mtROS in the execution of death (Varanita et al., 2015). IF1 has amply demonstrated its anti-apoptotic function both “*in vitro*” (Formentini et al., 2012; Faccenda et al., 2013; Sanchez-Arago et al., 2013b) and “*in vivo*” (Formentini et al., 2014; Santacatterina et al., 2016; Formentini et al., 2017) and is known to affect the activity and assembly of the ATP synthase and of other respiratory complexes (Campanella et al., 2008; Formentini et al., 2014; Mourier et al., 2014; Santacatterina et al., 2016; Cuillerier et al., 2017) as well as OPA1 mitochondrial cristae remodeling (Faccenda et al., 2017). Thus, challenges ahead are to establish the links between the signaling pathways activated by mitohormesis and the targets in mitochondria that control cell death at structural and functional levels. Undoubtedly, the ATP synthase plays a fundamental role in this arena providing a target for therapeutic intervention.

## Author Contributions

AG-A and JC wrote the paper. All the authors read, contributed, and approved the final manuscript.

## Conflict of Interest Statement

The authors declare that the research was conducted in the absence of any commercial or financial relationships that could be construed as a potential conflict of interest. The handling Editor and reviewer GL declared their involvement as co-editors in the Research Topic, and confirm the absence of any other collaboration.
